# Role, Targets and Regulation of (de)nitrosylation in Malignancy

**DOI:** 10.3389/fonc.2018.00334

**Published:** 2018-09-04

**Authors:** Salvatore Rizza, Giuseppe Filomeni

**Affiliations:** ^1^Redox Signaling and Oxidative Stress Research Group, Cell Stress and Survival Unit, Center for Autophagy, Recycling and Disease, Danish Cancer Society Research Center, Copenhagen, Denmark; ^2^Department of Biology, University of Rome Tor Vergata, Rome, Italy

**Keywords:** ADH5, GSNOR, nitric oxide, NOS, nitrosylation, cancer, FAK1, HIF-1α

## Nitric oxide involvement in cancer

**Figure 1:**
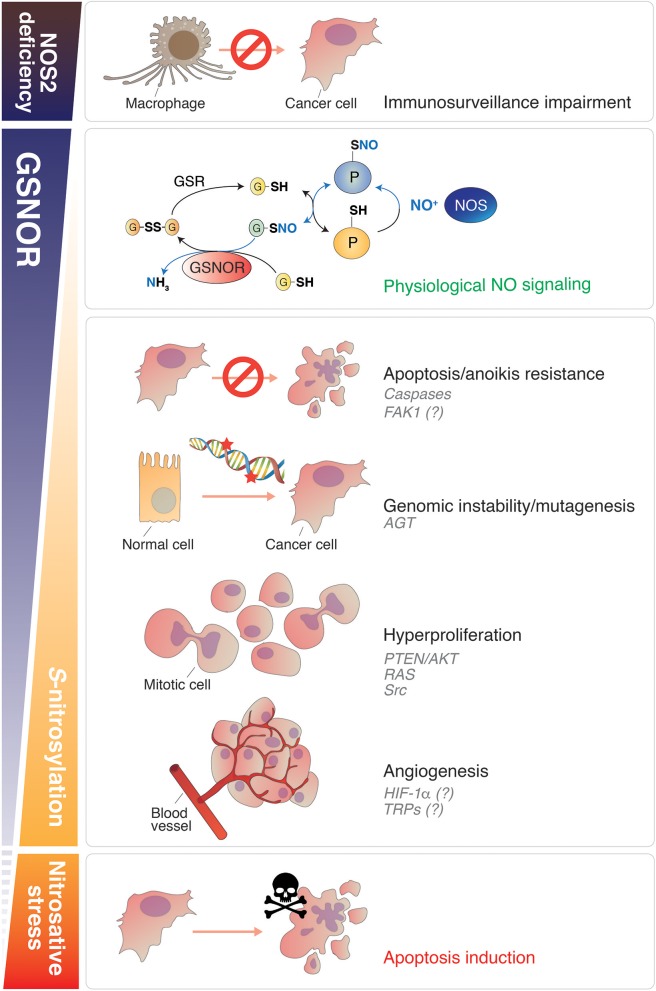
Roles of NO signaling and protein denitrosylation in cancer. Nitric oxide plays different roles in cancer biology depending on its concentration. GSNOR is the main cellular denitrosylase. Counteracting the effects induced by NOS, GSNOR finely modulates protein *S*-nitrosylation (second panel from the top), which is establishing as the main posttranslational modification underlying NO bioactivity. A disbalance in NO signaling can promote tumor induction, survival and progression. NOS2 deficiency impairs the capability of macrophages to kill cancer cells (Top). Conversely, in conditions of normal (or induced) NOS activity, GSNOR decrease has been linked to many cancer hallmarks, such as: (i) apoptosis and anoikis resistance (due to caspases and, reasonably, FAK-1 *S*-nitrosylation); (ii) genomic instability (DNA repair impairment, due to AGT *S*-nitrosylation and degradation); (iii) cells hyperproliferation (*via* the NO-mediated activation of oncoproteins, such as AKT, RAS, and Src); (iv) angiogenesis (putatively regulated by HIF-1α and TRPs *S*-nitrosylation). Extreme nitrosative stress conditions—induced, for instance, by NOS overexpression or by the use of NO-donors—activate cell death and are implemented (or physiologically activated in macrophages) to destroy cancer cells (Bottom). NO, nitric oxide; GSNOR, *S*-nitrosoglutathione reductase; NOS, nitric oxide synthase; FAK1, focal adhesion kinase 1; AGT, *O*^6^-methylguanine-DNA methyltransferase; HIF-1α, hypoxia-inducible factor-1α; TRP, Transient receptor potential channel.

Nitric oxide (NO) is a free radical that can target cellular biomolecules directly, or by means of the activity of its metabolites (RNS) generated upon reaction with transition metals (e.g., NO^+^), oxygen (e.g., N_2_O_3_), or superoxide (ONOO^−^). For instance, it is well-documented that NO and RNS affect DNA integrity and mitochondrial physiology, this leading to genetic mutations (1) and damage to the mitochondrial respiratory chain (2, 3), respectively. Processes ranging from apoptosis, angiogenesis, immunity, and neuronal physiology, all show seemingly contradictory behavior in response to NO. Indeed, the relevance of the steady-state NO concentrations represents a key determinant of its biological function. In support to this assumption, it has been demonstrated that cGMP-mediated processes occur at the low n*M* range, whereas higher NO concentrations cause protein kinase B (PKB)/Akt phosphorylation; stabilization of hypoxia inducible factor (HIF)-1α; phosphorylation of p53 and, at the μ*M* range, they can generate detrimental conditions usually referred as to nitrosative stress (Figure 1). Likewise, in tumor biology, it is now commonly accepted that high NO concentrations mediate apoptosis and cancer growth inhibition, whereas (relatively) low concentrations usually promote tumor growth and proliferation, this supporting the nature of “doubled-edged sword” molecule for NO (4, 5). This dichotomy originates from the observations that the inducible form of NO synthase (iNOS or NOS2) was implicated in the macrophage-mediated tumor killing process (6, 7) (Figure 1). NOS2^−/−^ mice develop intestinal tumors (8), thereby substantiating the protective role of NOS2 within host defense mechanisms (9, 10). In accordance, a growing body of evidence pointed out that NO-releasing drugs can be toxic for cancer cells.

On the other hand, low rate of NO production can promote tumor growth rather than killing. In line with this assumption, the overexpression of NOS isoforms has been detected in a wide range of human tumors. In particular, NOS2 has been found to be upregulated in melanoma, estrogen receptor-negative (ER^−^)-breast cancer, as well as in pancreatic, cervical liver and ovarian cancers (10). Moreover, NOS2 seems to be involved in maintaining physiologically relevant levels of NO to sustain the progression phase of carcinogenesis; mainly it is required to promote angiogenesis and to enhance the ability of cancer cells to counteract nutrient paucity in solid tumors and to metastasize (11, 10). NOS2 is also overexpressed in glioma stem cells, and its activity is required for the expression of the cell cycle inhibitor cell division autoantigen-1 (CDA1), which sustains growth and tumorigenicity (12). NOS2 has been also found to be upregulated in hepatocellular carcinoma (HCC), and is often increased in the hepatocytes of patients with chronic hepatitis and alcoholic cirrhosis, conditions that predispose to HCC (13–15). Notwithstanding all these lines of evidence, investigations on NOS2^−/−^ mice, in spontaneous and fibrosis-associated models of HCC, reveal little effect of NOS2-derived NO on hepatocarcinogenesis (16), meaning that other players are also involved.

### *S*-nitrosylation and cancer

Redox signal underlying both pro-survival and death pathways, is a molecular information transduced by means of reactive cysteine residues that can undergo *S*-hydroxylation (*SOH*), upon reaction with ROS (i.e., H_2_O_2_) or *S*-nitrosylation (*SNO*), the posttranslational modification induced by NO, which is now emerging to underlie NO bioactivity (17). In the presence of a sulfhydryl group in their close proximity, both these modifications can resolve in a more stable disulfide bridge (*S*-thiolation, *SS*) (18–20). Actually, it has been recently questioned whether *S*-nitrosylation—given its nature of instable posttranslational modification—is able to convey the NO-mediated signal, or just acts as mere intermediate for disulfide bridge formation (21). Whatever is the end effector (if directly the SNO group or, indirectly, the SS adduct), the extent of *S*-nitrosylation is determined by a delicate balance between: (i) the rate of NO production, which is catalyzed by NOSs (22, 23), (ii) the activity of a recently discovered class of enzymes termed nitrosylases (24, 25), and (iii) the efficiency of SNO removal, that is mediated by denitrosylases. *S*-nitrosoglutathione reductase (GSNOR) represents the prototype of this class of oxidoreductases and, so far, the only denitrosylase able to completely reduce NO moiety, reason why it has been also termed GSNO *terminase* (26–28). Notwithstanding current literature offers still conflicting lines of evidence about the role of NOS/NO system in cancer biology, even less is known on the role played by GSNOR and denitrosylation. In this scenario, it has been reported that GSNOR-ablated (GSNOR-KO) mice show predilection to hepatocellular carcinoma (HCC) in association with *S*-nitrosylation and proteasomal degradation of the DNA damage repair enzyme *O*^6^-alkylguanine-DNA alkyltransferase (AGT) (29). As a result, the repair of carcinogenic *O*^6^-alkylguanines is significantly impaired with a consequence increase in tumorigenesis (29, 30). Analyses performed on human HCC patients showed a significant decrease of GSNOR protein levels and activity in the 50% of cases (30), arguing for a functional link between GSNOR-dependent *S*-nitrosylation and HCC. Although this evidence supports a driving role for GSNOR and excessive *S*-nitrosylation in HCC ontogenesis, it is still unknown whether they are also implicated in the other phases of carcinogenesis, e.g., tumor promotion and progression (31).

In this regard, it has been published that GSNOR deficient HCC cells have a compromised mitochondrial electron transport chain characterized by the upregulation of succinate dehydrogenase (*SDH*), likely as an adapting response to the general impairment of the mitochondrial respiratory machinery (32) derived from excessive nitrosative stress. The hyper-nitrosylation of the mitochondrial chaperone TNF receptor–associated protein 1 (Trap1) has been identified as the molecular event responsible for such a rearrangement and, in turn, for the enhanced sensitivity of GSNOR-downregulating HCC to SDH-targeting mitochondrial drugs (32). Nevertheless, it is worth to note that the mean size of GSNOR-deficient tumor xenografts is larger (approximately the double) than parental (GSNOR-proficient) HCC (32), suggesting that excessive *S*-nitrosylation arising from GSNOR loss, might promote tumor progression and growth *in vivo*. This hypothesis finds support in a recent study correlating GSNOR downregulation with HER2^+^ breast cancer resistance to trastuzumab and poor patient prognosis (33). Altogether, these pieces of evidence argue for a new role of GSNOR in malignancy and resistant phenotypes of breast cancer. However, no evidence about the molecular mechanisms underneath has been provided so far.

### Known and supposed targets of *S*-nitrosylation in aggressive cancer

Based on what above reported, it is plausible that impairments of denitrosylation capacity (e.g., upon GSNOR deficiency) modulates the function/activity of oncoproteins susceptible to *S*-nitrosylation. These defects, more specifically than a general increase of NO production (that could impact on a plethora of different targets and to a different extent) might account for a deregulated NO-signaling in carcinogenesis. This hypothesis is further sustained by a very recent study indicating that GSNOR-deficiency (and excessive *S*-nitrosylation deriving from it), is a condition associated with aging (34, 35), which represents a major risk factor for cancer development. Actually, cancer might count as an aging disease, and shares with aging some common features (e.g., genomic instability, telomere shortening, oxidative stress, deregulation of nutrient sensing) that, indeed, characterize both disorders (36).

Besides those previously mentioned, and others well documented to play a role in apoptosis (e.g., p53, Bcl2, and Fas), many oncoproteins have been discovered in the last decades to undergo *S*-nitrosylation. The modification of oncoproteins and tumor suppressors by NO—independently on the effects induced, whether gain- or loss-of-function (23, 37)—is emerging as a critical phenomenon associated with neoplastic transformation. Some of these NO-modified oncoproteins participate to signal transduction and are found mutated or modulated in cancer. Within this class of proteins in which *S*-nitrosylation has been identified as pro-oncogenic modification, we can list: (i) the GTPase Ras (nitrosylated at Cys118) (23, 38), which underlies cancer cell growth downstream of receptor-associated tyrosine kinases; (ii) the phosphatase and tensin homolog PTEN (nitrosylated at Cys83) (39), which regulates the levels of phosphatidyl inositole-3-phosphate/Akt-dependent pathway; (iii) the protein kinase c-Src, which represents one of the master regulators of tumor proliferation, invasion and metastatic phenotype, and has been found to be nitrosylated at Cys498 (40) (Figure 1). Interestingly, this residue is conserved throughout the Src family of protein tyrosine kinases (SFKs) and, at least for other two members, i.e., Yes and Fyn, has been also reported to stimulate their activation (40). Focal adhesion kinase 1 (FAK1) is also comprised in the SFKs family. It is phosphorylated upon integrin engagement in a Src-dependent or independent (auto-phosphorylation) fashion, thus initiating multiple downstream signaling pathways responsible for aggressive and metastatic phenotype (e.g., resistance to anoikis and cell migration) (41). Similarly to Src, Yes and Fyn have been reported to act as FAK1-interacting kinases and to be involved in FAK1 activation as well (42, 43). Notwithstanding this tight relationship, triple-KO cells in which Src, Yes, and Fyn expression is suppressed (*SYF* cells), still show phospho-active levels of FAK1 upon treatment with NO donors (40). This unexpected evidence clearly indicates that *S*-nitrosylation of Src, Yes, and Fyn is dispensable for NO-driven phosphorylation of FAK1 and, interestingly, suggests that FAK1 might represent a direct target of *S*-nitrosylation (Figure 1), with this modification driving its oncogenic function.

Another oncoprotein, which has been identified to be crucial in cancer cell survival and growth, especially under low-oxygen tension (hypoxia), is HIF-1α. HIF-1α deregulation has been deeply implicated in different aspects of cancer biology, such as angiogenesis, cell resistance, and tumor invasion (44–47). From a metabolic point of view, HIF-1α aberrant activation underpins the so-called “Warburg effect”: the preferential glycolytic consumption of glucose in cancer cells, which takes place also under normal oxygen tension. HIF1α has been found nitrosylated at Cys533 (48), with this being relevant in stroke and cardiovascular disease. However, if S-nitrosylation might somehow induce HIF1α oncogenic activity still remains neglected (Figure 1) and would deserve to be investigated in the future.

Among the various classes of proteins that have been identified in the last decades as being activated by *S*-nitrosylation, the transient receptor potential (TRP) ion channels (49), which represent a huge family of proteins underpinning, among others, warm, taste, and pain sensory transduction, are worth to be mentioned. Besides their well-documented role in the nervous system as mediators of sensations, in the last years it is emerging that many TRPs, such as those belonging to the “melastatin” (TRPM), “vanilloid” (TRPV), and “ankyrin” (TRPA) subfamilies, are overexpressed in many cancer types, this being pivotal for calcium signaling-dependent control of tumor-promoting processes, e.g., vascularization and metastasis (50, 51). In particular, it has been proposed that, by modulating intracellular Ca^2+^ concentrations, TRPs are deeply involved in tumor initiation, progression and resistance (52). In this context, it has been very recently found out that TRPA1 is upregulated in breast and lung cancer downstream of the activation of Nrf2, the master regulator of antioxidant response, this conferring non-canonical resistance to tumor cells against oxidative stress and ROS-producing chemotherapeutics (53). Many other observations argue for TRPs inhibition being a promising tool to eradicate cancer (54–56). However, notwithstanding the evidence that *S*-nitrosylation interferes with TRPs activity and calcium signaling, to date there's still no indication supporting a direct involvement of TRPs *S*-nitrosylation in carcinogenesis (Figure 1). Mostly, there's still no study aimed at understanding whether TRPs targeting on nitrosylable cysteines might represent a novel line of intervention in cancer treatment.

### Putative mechanisms that affect denitrosylation in cancer

The above reported evidence points out that defects in GSNOR expression and denitrosylation are pivotal for sustaining the tumorigenic effects of NO, namely, its role in the progression phase of cancer (31). A recent report on the epigenetic regulation of GSNOR might be of help to understand how this condition can be established in cancer. In particular, it has been demonstrated that *GSNOR* expression is controlled by the activity of the demethylase Ten-eleven translocation protein 1 (Tet1), a member of the 2-oxoglutarate–dependent dioxygenases that regulates transcription by removing methyl groups from CpG islands located in the promoter regions of genes (34). Remarkably, Tet1 expression has been found to be reduced in a wide range of solid cancers, such as melanoma, prostate, lung, and liver tumors (57, 58)—where also GSNOR mRNA seems to be downregulated—and to correlate with advanced cancer stage, nodal metastases, and poor survival rate in breast cancer patients (59). These lines of evidence suggest that GSNOR might be epigenetically downregulated in aggressive cancer as a consequence of Tet1 reduction, thus providing a new link between epigenetics and redox signaling. This hypothesis can be even extended to further mechanisms of epigenetic regulation. Indeed, given the complex structure of GSNOR mRNA, it has been proposed that GSNOR expression might be also regulated *via* microRNAs (miRs) (27). However, no putative miRs, able to target GSNOR transcript, has been so far identified to be upregulated in cancer, or hypothesized acting as additional modulators of *S*-nitrosylation.

## Conclusion

The role of GSNOR-mediated denitrosylation in carcinogenesis has been capturing the interest of many researchers working on cancer biology, as many lines of evidence indicate that this process is frequently deregulated in cancer cells. In this article, we have tried to summarize what has been discovered in the last years and provide some hints on possible aspects that are still overlooked. Understanding how GSNOR expression is deregulated in may cancer histotypes, as well as the mechanisms underlying the modification of new protein targets involved in cancer resistance and aggressiveness, are, indeed, issues that deserve to be investigated in the future, since they could set the stage for new anticancer approaches interfering with the redox adaptation distinctive of many cancer cells.

## Author contributions

GF conceived the paper. GF and SR wrote the paper. SR drew the figure.

### Conflict of interest statement

The authors declare that the research was conducted in the absence of any commercial or financial relationships that could be construed as a potential conflict of interest.
